# The susceptibility assessment of debris flow hazards based on the game theory combination weighting-normal cloud model

**DOI:** 10.1371/journal.pone.0310775

**Published:** 2024-09-26

**Authors:** Xiu-Tao Yang, Xin-Bao Gu

**Affiliations:** 1 Henan Planning Design & Research Institute Co. Ltd., Zhengzhou, Henan, China; 2 School of Civil Engineering, Nanyang Institute of Technology, Nanyang, Henan, China; ICIMOD: International Centre for Integrated Mountain Development, NEPAL

## Abstract

The susceptibility evaluation of debris flow has excellent significance for people’s life and property safety. The game theory combination weighting-normal cloud model is applied to evaluate its susceptibility in the paper. Firstly, the length ratio of the supply segment (X_1_), the longitudinal slope of the main ditch (X_2_), the slope of the mountain (X_3_), watershed area (X_4_), the relative difference (X_5_), the vegetation coverage (X_6_), as well as the daily maximum rainfall (X_7_) are adopted as the assessment index; the game theory combination weighting-normal cloud model is introduced. Secondly, the normal cloud model of specific debris flow hazards is established; the weight coefficient of each index is calculated using the game theory combination weighting method, and the membership degree of each index is determined using the cloud model; finally, conclusions are drawn that the results obtained by the suggested method are consistent with the actual investigation for eight different gullies. Its accuracy reaches 100% for the proposed method, which is higher than the results from the gray extension model (62.5%); its assessment results predict the susceptibility level of debris flow hazards accurately and further determine the susceptibility grade ranking for different gullies at the same level. Therefore, it can provide a new method and thought for the susceptibility assessment of debris flow hazards in the future.

## 1. Introduction

Debris flow is a common geological disaster phenomenon in the loess area [1]. Under heavy rainfall, collapse and landslide instability provide material sources for debris flow and increase the probability of occurrence [2, 3]. According to recent statistical data, the debris flow hazards distributed in 31 provinces, cities, and autonomous regions in China, including 950 towns. Active area of Debris flow has reached 4.3 million km^2^, and there are approximately 80,000 debris flow activities in China, of which 10,000 are very serious [4]. In recent years, the annual economic losses caused by debris flow have arrived 1.5 billion and 2 billion yuan, the death toll has reached 250~500 people, and the safety of people’s lives and property has been seriously affected [5]; therefore, it is necessary to perform an accurate susceptibility evaluation for the influence degree and scope of debris flow disaster [6].

Many countries’ researchers have suggested models to assess the susceptibility level of debris flow hazards. At present, there are many susceptibility assessment methods of debris flow, such as fuzzy comprehensive evaluation [7], analytic hierarchy process [8], artificial neural network [9], standard way, and extension method [10]. For example,Wang et al. [11] evaluate the danger of debris flow using the method of fuzzy mathematics; Wang et al. [12] applied the multi-level fuzzy evaluation method to assess the susceptibility level of debris flow; Zhao et al. [13] used the method of neural network to determine the susceptibility of debris flow; Wei et al. [14] used the concept of information in the information theory and the process of fuzzy evaluation to distinguish the susceptibility level of debris flow; Menoni et al. [15]combined the disaster management function with empirical qualitative approach to analyze the vulnerability of debris flow disasters in the Alps; O’ Brien [16] used the finite element analysis software to describe the shape of debris flow accumulation area, and divided debris flow risk zones. With the application of GIS technology and the development of computer, more and more machine learning algorithms are applied to susceptibility assessment of debris flow hazards; for instance, support vector machine [17], decision trees [18] and random forests [19] have been successfully used in the study of geological hazards; and Walsh et al. [7] carried out a GIS-based visualization of debris flow patterns; Perotto et al. [20] evaluated the geomorphology disaster and population vulnerability with the support of GIS technology; the above analysis method represented by machine learning can objectively express the non-linear mapping relationship between the disaster-generating condition and the danger of debris flow, the interference of artificial subjective factors is avoided, and the evaluation result is more accurate.

Although the above method promotes the susceptibility assessment theory of debris flow enormously, some things still need to be improved [5]. For example, complex calculation processes should have addressed randomness and low efficiency et al. [6, 21]. To overcome the insufficiency of the above methods, the game theory combination weighting-normal cloud model is introduced to assess the susceptibility level of debris flow hazards; the technique applies the game theory combination weighting method to determine the weights of each evaluation index, and then the normal cloud method is used to calculate the membership degree of each index. Finally, a synthetic membership degree is constructed, and the susceptibility level of debris flow hazards is determined. Its results have higher reliability and efficiency than the above method, so applying the suggested model to assess the susceptibility level of debris flow is crucial.

The paper is organized as follows: in Section 2, the engineering overview is introduced at first; in Section 3, theory and methodology based on the game theory combination weighting-normal cloud model are presented; in Section 4, the assessment model of the debris flow hazards is constructed, and the assessment results are analyzed; in Section 5, conclusions are drawn.

## 2. Engineering overview

**Fig 1:**
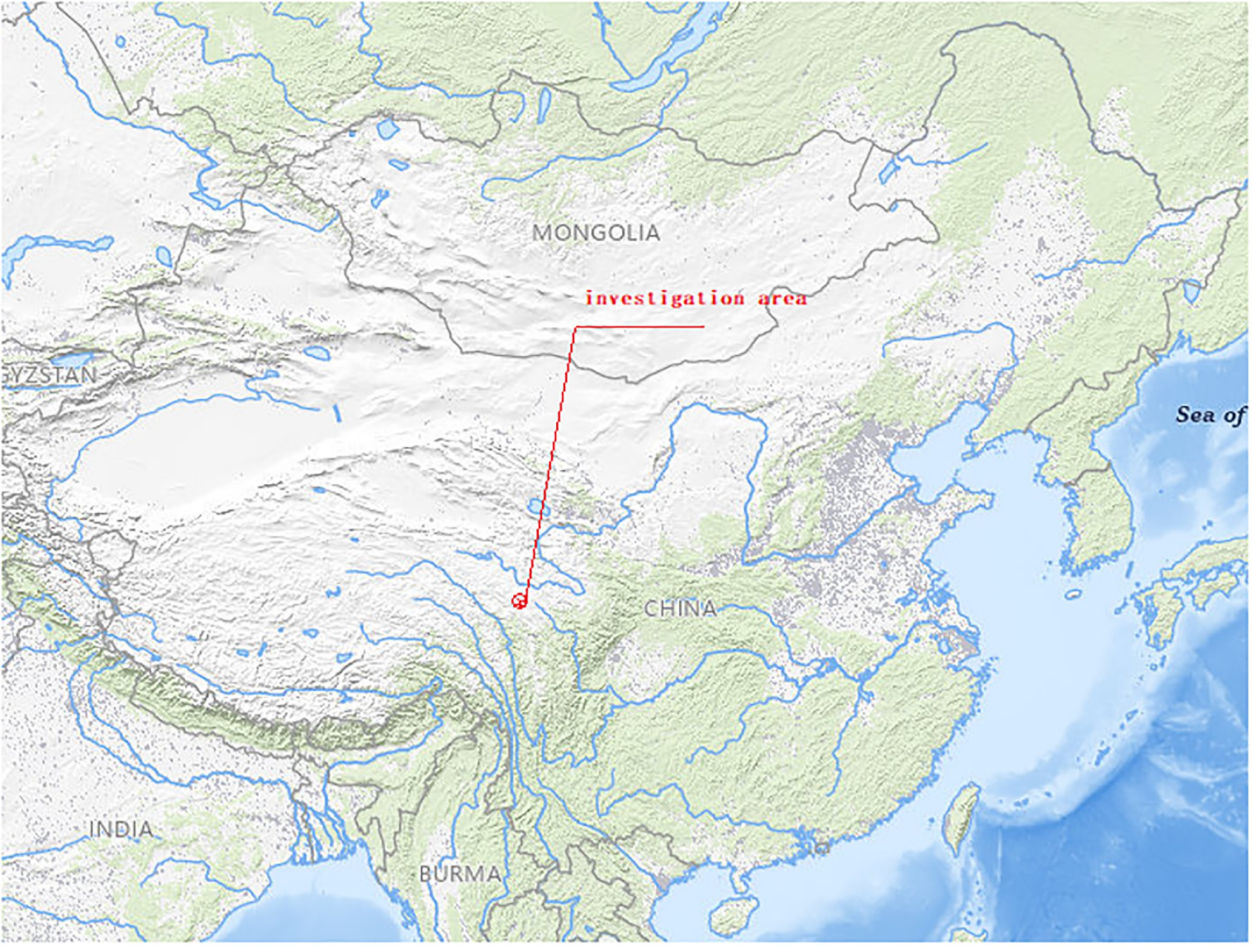
Location map of investigation area. (https://www.usgs.gov/tools/national-map-viewer).

The study area is located in Lintao County, Gansu Province, China (it is plotted in Fig 1), and it is the lower reaches of the Tao River watershed southwest of the Loess Plateau. The investigation area is mainly covered by loess with mudstone, sandstone and Gariton granite. According to the trend of debris flow gullies, most of them are distributed in the east-west direction, and the terrain is gradually reduced from the source area to the direction of the Tao River; the formation area and the circulation area are located in the Zhongshan landform, and the debris flow ditch is long. Because most debris flow gullies are located at 1750–2500 m above sea level, the highest elevation of the Dongjialing is 2577.7 m above sea level, the lowest edge of Tao River is 1735 m above sea level; the elevation in the area is 1850-2450m, the relative height difference is 100~600m. Due to the significant height difference (plotted in Fig 1) and abundant loose slope deposits, the debris flow gully at both sides of the mountain body is widely distributed. After long-term weathering and rainfall erosion, the rock mass is easy to slide down from both sides of the debris flow gully, and the material resource is formed.

In the study area, the slope is significant; it is usually 20° ~ 40°, the gully on the slope surface is relatively developed, the depth is generally 30 ~ 70 m, and the width is mostly 30 ~ 50 m. On the west bank of Tao River, the slope on both sides of the debris flow gully is steep, even though some slope angles arrive at more significant than 40°. Therefore, the water can quickly form in the gullies during precipitation. When the amount of water reaches a certain extent, it will promote the occurrence of debris flow geological hazards.

## 3. Methodology

### 3.1 The combination weighting method

The standard weight calculation methods are divided into subjective, objective, and combination weights. Combination weighting is a common method; two or three kinds of subjective and objective weights are combined to get the comprehensive weight, which can reduce the error caused by a single method to a certain extent [21, 22]. In this study, the entropy weight and CRITIC methods are applied to calculate the weights of the indexes, and the combination weights are obtained using game theory.

(1) The Entropy method

The entropy weight method is an objective weighting method to determine the weight coefficient according to each evaluation index’s degree of information utility value [23, 24]. The entropy weight method can reflect the degree of discreteness among the index data.

Its calculative process is listed as follows:

① constructing the original matrix of assessment index *X*

Assuming that there are *m* evaluation indexes and *n* evaluation objects, *x*_*ij*_ is the corresponding value of *ith* assessment index at the *jth* assessment object; then its origin assessment matrix can be expressed as:

$$X={\left(x_{ij}\right)}_{m\times n}\left(i=1,2,\ldots ,m;j=1,2,\ldots ,n\right)$$ (1)

② Normalization and forward processing

Due to the different types of indicators and dimensional differences, in order to rule out the impact of these differences, the dimensionless processing to each index need be performed, they are expressed as:

$$Y=\left({\text{y}}_{ij}\right)\left(i=\right)1,2,\ldots ,m,j=1,2,\ldots .,n)$$ (2)

The positive indicators are:

$$y_{n}=\frac{x_{ij}-\text{min}\left(x_{ij}\right)}{\text{max}\left(x_{ij}\right)-\text{min}\left(x_{ij}\right)}$$ (3)

The negative indicators are:

$$y_{n}=\frac{\text{max}(x_{ij})-x_{ij}}{\text{max}\left(x_{ij}\right)-\text{min}\left(x_{ij}\right)}$$ (4)

Where, *y*_*ij*_ is the standard value of *ith* assessment index at the *jth* assessment object.

③ Calculating the information entropy of *ith* assessment index [4]

$$h_{i}=\frac{1}{\text{ln}\;n}\sum_{j=1}^{n}e_{ij}\;\text{ln}\;e_{ij}$$ (5)

$$e_{ij}=\frac{y_{ij}}{\sum_{j=1}^{n}y_{ij}}$$ (6)

④ the calculation of weights *ω*_li_

$$\omega_{\text{1i}}=\frac{1-h_{i}}{m-\sum_{i=1}^{m}\;h_{i}}$$ (7)

Where, $0<\omega_{i1i}\leq 1,\sum_{i=1}^{m}\;\omega_{1i}=1,i=1,2,\ldots ,m.$

(2) the CRITIC method

CRITIC(Criteria importance through inter criteria correlation) is an objective weighting method proposed by Diakoulaki, which synthetically measures the index weight by calculating the variability and conflict of the index. Its calculative procedure is listed as follows [25]:

① it is assumed that there are m estimated object and n assessment index, they construct a matrix *A* = (*a*_*ij*_)_*m*×*n*_, where, *i* = 1,2,…,*m*; *j* = 1,2,…,*n*.

② the matrix *A* is standardized based on Z-score method,its expression is shown as follows:

$$a_{ij}^{*}=\frac{a_{ij}-\bar{a_{j}}}{s_{j}}(i=1,2,\ldots ,m;j=1,2,\ldots ,b)$$ (8)

Where, $\bar{a_{j}}=\frac{1}{a}\sum_{i=1}^{m}a_{ij}$, sj=∑i=1maij−aj¯a−1, $\bar{a_{j}}$ and *s*_*j*_ are respectively mean value and standard deviation of the *jth* assessment index.

③ calculate the coefficient of variation of different indexes, it can be calculated as follows:

BYj=sjaj¯(j=1,2,…n) (9)

Where, *BY*_j_ is the variation coefficient of the *jth* index.

④ the coefficients of correlation are calculated based on the standardization matrix A*, its expression is listed as follows: *X* = (*r*_*kl*_)_*n*×*n*_(*k* = 1,2,…, *n*, *l* = 1,2,…, *b*), *r*_*kl*_ is the coefficients of correlation between the *kth* and *lth* index,and:

$$r_{kl}=\frac{\sum_{i=1}^{m}\left(a_{ik}-\bar{a_{k}}\right)\left(a_{il}-\bar{a_{l}}\right)}{\sqrt{\sum_{i=1}^{m}{\left(a_{ik}-\bar{a_{k}}\right)}^{2}}\sqrt{\sum_{l=1}^{m}{\left(a_{l}-\bar{a_{l}}\right)}^{2}}}\;\left(r_{kl}=r_{lk};k=1,2,\ldots ,m,l=1,2,\ldots ,m\right)$$ (10)

Where, *a*_*ik*_ and *a*_*il*_ are respectively the standard value of measured values at the *kth* and *lth* index for the *ith* assessment object in the standardization matrix A*; $\bar{a_{k}}$ and $\bar{a_{l}}$ are respectively the mean of standard value of measured values at the *kth* and *lth* index in the standardization matrix A*.

⑤ the calculation of the quantitative coefficient about degree of independence for different assessment indexes

Its expression is shown as follows [26]:

$$\eta_{j}=\sum_{k=1}^{n}\left(1-\left|r_{kj}\right|\right)\left(j=1,2,\ldots ,n\right)$$ (11)

⑥ The quantitative coefficients of the comprehensive information and the degree of independence of each index are solved as follows:

$$C_{\text{j}}=BY_{j}\sum_{k=1}^{n}\left(1-r_{kj}\right)\left(j=1,2,\ldots ,n\right)$$ (12)

⑦ The determination of the weight of each evaluation index, it can be expressed as:

$$\omega_{\text{j}}=\frac{C_{j}}{\sum_{j=1}^{n}\;C_{j}}\left(j=1,2,\ldots ,n\right)$$ (13)

(3) the combination weighting method of the game theory

Based on game theory, the combination weight *ω* is obtained by combining the entropy weight method with the CRITIC method. Its procedure is correlated as follows [27]:

① The weight sets *ω*_1_ and *ω*_2_ were obtained by entropy weight method and CRITIC method. It is assumed that *a*_1_ and *a*_2_ are respectively linear combination coefficient, then weight sets *ω*_1_ and *ω*_2_ can be linearized as:

$$\omega =a_{1}\omega_{1}^{T}+a_{2}\omega_{2}^{T}$$ (14)

② According to the game theory, the linear combination coefficients *a*_1_ and *a*_2_ in formula (10) are optimized, they are expressed as follows:

$$\text{min}{\left\|a_{k}\omega_{k}^{T}-\omega_{k}\right\|}^{2}\;(k=1,2)$$ (15)

③ According to the differential properties of the matrix, the linear differential equation group for optimizing the first derivative condition of formula (15) is determined as:

$$\left[\begin{array}{cc} \omega_{1}\omega_{1}^{T} & \omega_{1}\omega_{2}^{T}\\ \omega_{2}\omega_{1}^{T} & \omega_{2}\omega_{2}^{T} \end{array}\right]=\left[\begin{array}{c} \omega_{1}\omega_{1}^{T}\\ \omega_{2}\omega_{2}^{T} \end{array}\right]$$ (16)

④ The optimal combination coefficients *a*_1_ and *a*_2_ were obtained by formula (16), the normalization process is obtained as a1*=a1a1+a2, a2*=a2a1+a2,then based on the game theory, the comprehensive weight *ω* can be obtained as:

$$\omega =a_{1}^{*}\omega_{1}+a_{2}^{*}\omega_{2}$$ (17)

### 3.2 The normal cloud model

The cloud model is defined as: *x*, *E*, *D* is assumed as a common quantitative set, *E* is called as the domain; where, *x* ∈ *E*, *D* is the qualitative conception in domain *E*. For the random research object *x* in the domain *E*, there still exists a random number with the stable tendency *u*(*x*) ∈ [0, 1], then *u*(*x*) is called as the membership degree of *x* corresponding to *D*, or it is called as the definitive degree. The distribution of definitive degree in the domain *E* is called as the membership cloud. If *x* meets with *x* ∼ *N*(*Ex*, *En*’^2^), and *En*’ ~ *N*(*En*, *He*^2^), and then *u*(*x*) can be expressed as:

$$u\left(x\right)=\text{exp}\left[-\frac{{\left(x-Ex\right)}^{2}}{2E{n'}^{2}}\right]$$ (18)

Where, the distribution definitive degree *u*(*x*) in the domain *E* is also called as normal cloud or Gauss cloud. The expectation *Ex*, the entropy *En* and hyperentropy *H*_*e*_ are respectively applied to represent the digital features in the cloud model. *Ex* can represent the point of certain conception in the domain; *En* reflects the accepting range of conception; *H*_*e*_ demonstrates the uncertainty of Entropy, its magnitude reflects the thickness of cloud drop. They can respectively be expressed as:

$$Ex=\frac{c^{+}+c^{-}}{2}$$ (19)

$$En=\frac{c^{+}-c^{-}}{6}$$ (20)

$$H_{\text{e}}=k$$ (21)

Where, *c*^+^ and *c*^−^ are respectively the upper and lower bounds corresponding to the grade standard of specific index. The hyperentropy *H*_*e*_ can be selected a proper constant *k*, *k* is set as 0.01 in the investigation.

## 4. The construction of assessment model

### 4.1 The determination of the evaluation index

For the selection of evaluation indicators, it should have a clear physical significance, mutual independence, and easy access to quantitative processing. According to the above principles and in combination with the actual investigation in the study area, the critical seven factors are selected as the assessment index of debris flow; the evaluation factors, respectively, are:

(1) the length ratio of the supply segment (*X*_*1*_)
  The length ratio of the supply segment represents the ratio between the accumulative length of the supply section and the length of the main ditch; it reflects the range and amount of sediment supply. The ratio is more excellent, the supply condition is better, and the susceptibility degree is higher.
(2) the longitudinal slope of the main ditch(*X*_*2*_)
  It means that the potential energy of debris flows directly; when its magnitude becomes more incredible, the susceptibility degree of debris flow is higher.
(3) the slope of the mountain(*X*_*3*_),
  It reflects the magnitude of the potential energy of debris flow indirectly. When its magnitude becomes more remarkable, the potential energy of debris flow is greater; the susceptibility degree is higher.
(4) watershed area(*X*_*4*_)
  It demonstrates the sand production and confluence state; its magnitude and susceptibility degree are greater.
(5) the relative difference(*X*_*5*_)
  It is defined as the maximum relative height difference in the whole watershed. It means that when its magnitude becomes more remarkable, the potential energy of the sediment is more significant, and the susceptibility degree of debris flow is higher.
(6) the vegetation coverage(*X*_*6*_)
  It is depicted as the ratio of forest cover area and total watershed area; its magnitude is greater, and the susceptibility degree of debris flow is lower.
(7) the daily maximum rainfall (*X*_*7*_).
  It reflects the kinetic energy of debris flow indirectly. When its magnitudes become more excellent, the susceptibility degree is higher.

The eight gullies are selected as the typical debris flow gully; their numbers and names are:1(Chubula gully), 2 (Zhangjia gully), 3(Tashi gully), 4(Zhan gully),5(Quan gully), 6(Huozuguang gully),7(Zhangnaila gully) and 8(Sha gully).

This paper shows the monitoring data of indexes in eight debris flow gullies in Table 1. According to the characteristics of engineering research, the assessment factors of debris flow susceptibility are classified into specific grade criteria, as shown in Table 2. They are classified into four grades: I(low danger, II (medium danger), III (high danger), and IV (extreme danger).

**Table 1:** The monitoring values of each evaluation factor.

Index name	X_1_	X_2_	X_3_	X_4_	X_5_	X_6_	X_7_
1	53	2	45	21.6	625	50	143.8
2	65	13	42	2.5	620	40	64.7
3	42	14	55	1.3	260	25	44.5
4	72	4	47	11	460	7	143.8
5	53	8	44	23	370	8	143.8
6	65	14	55	0.6	354	70	64.7
7	62	14	45	0.7	430	65	64.7
8	68	17	38	0.9	620	48	44.5

**Table 2:** Index classification of debris flow susceptibility.

Assessment index	The Vulnerability level			
	I	II	III	IV
X_1_	≤10	10~30	30~60	≥60
X_2_	≤3	3~6	6~12	≥12
X_3_	≤15	15~25	25~32	≥32
X_4_	≤5	5~10	10~100	≥100
X_5_	≤100	100~300	300~500	≥500
X_6_	≥60	30~60	10~30	≤10
X_7_	≤25	25~50	50~100	≥100

### 4.2 The construction of the evaluation frame

**Fig 2:**
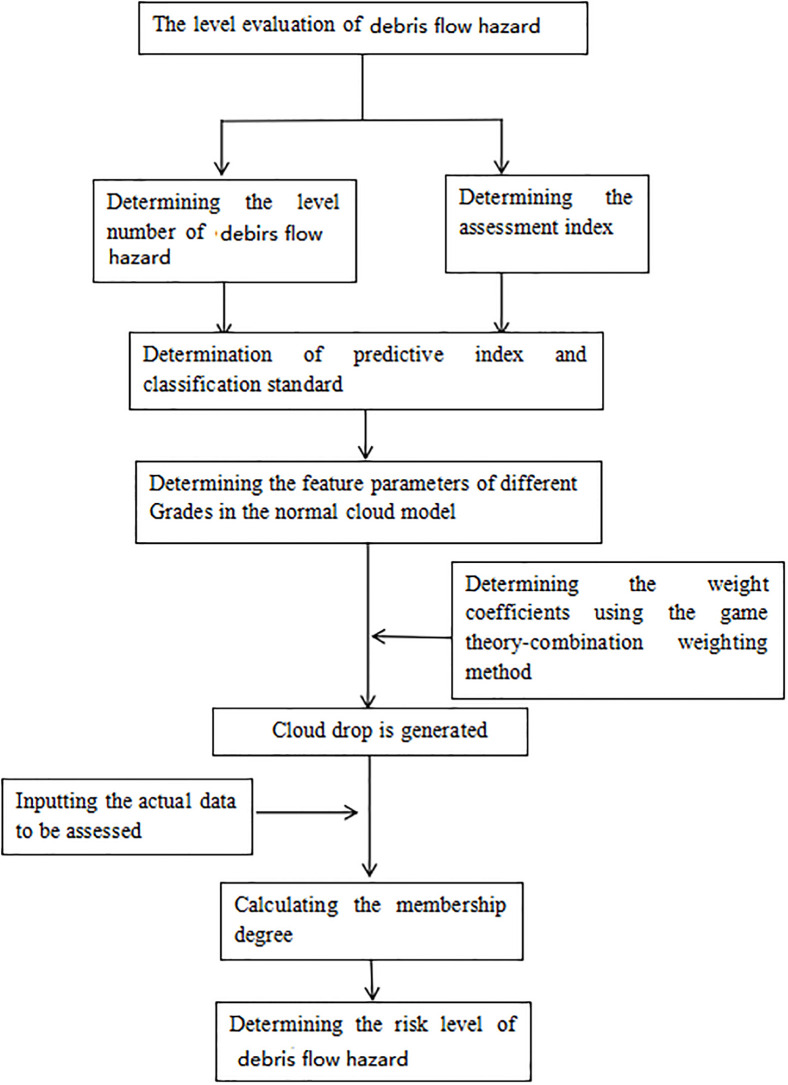
The flowchart of assessment frame.

The flowchart of the assessment frame is plotted in Fig 2. At first, the predicting index and corresponding intervals are determined, and then the sample datum’s weight calculation is performed using the game theory combination weighting method. Characteristic parameters *Ex*, *En* and *H*_*e*_ are calculated in the cloud model based on the classification interval of the assessment index. Finally, the synthetic membership degree *M* (shown in Eq (22)) can be obtained using the datum to be assessed and in combination with the weight of the assessment index. The final level of debris flow hazards can be determined according to the maximum certainty degree criterion.

$$M=\sum_{\text{i}=1}^{\text{n}}\;u_{i}\omega_{i}$$ (22)

### 4.3 Determining the weight coefficients

(1) calculating the weight coefficient *ω*_1_ based on the Entropy method

According to Eqs (1)–(7), and in combination with Table 1, the corresponding weight coefficient can be obtained:

$$\omega_{1}=\left[\begin{array}{ccccccc} 0.0106 & 0.116 & 0.0061 & 0.5702 & 0.0346 & 0.1658 & 0.0967 \end{array}\right]$$

(2) calculating the weight coefficient *ω*_2_ based on the CRITIC method

Based on Eqs (8)–(10), and in combination with Table 1, the coefficients of correlation can be calculated as:

$$r=\left[\begin{array}{ccccccc} 1 & 0.0667 & 0.4129 & 0.2688 & 0.4695 & 0.1761 & 0.0138\\ 0.0667 & 1 & 0.0166 & 0.8383 & 0.1742 & 0.4436 & 0.9397\\ 0.4129 & 0.0166 & 1 & 0.2003 & 0.7946 & 0.0697 & 0.132\\ 0.2688 & 0.8383 & 0.2003 & 1 & 0.1305 & 0.4978 & 0.9154\\ 0.4695 & 0.1742 & 0.7946 & 0.1305 & 1 & 0.2092 & 0.1172\\ 0.1761 & 0.4436 & 0.0697 & 0.4978 & 0.2092 & 1 & 0.5244\\ 0.0138 & 0.9397 & 0.132 & 0.9154 & 0.1172 & 0.5244 & 1 \end{array}\right]$$

According to Eq (11), the standard deviation of different columns is obtained as

$$\eta =\left[\begin{array}{ccccccc} 4.5923 & 3.521 & 4.374 & 3.1489 & 4.1049 & 4.0792 & 3.3577 \end{array}\right]$$

Similarly, according to Eqs (12) and (13), the weight of each evaluation index can be calculated as

$$\omega_{2}=\left(\begin{array}{ccccccc} 0.1461 & 0.1228 & 0.1478 & 0.131 & 0.1526 & 0.1498 & 0.1498 \end{array}\right)$$

(1) Calculating the combination weight

Based on the Eqs (14)–(17), and in combination with weight sets *ω*_1_ and *ω*_2_, the combination weight *ω* can be obtained as follows:

$$\omega =\left[\begin{array}{ccccccc} 0.0211 & 0.1165 & 0.0171 & 0.5362 & 0.0437 & 0.1646 & 0.1008 \end{array}\right]$$

### 4.4 The determination of digital features in the normal cloud model

Based on Table 2, and in combination with Eqs (19)–(22), the classification standard of normal cloud about debris flow is depicted in Table 3.

**Table 3:** The digital feature of cloud model.

Risk level	The digital feature	X_1_	X_2_	X_3_	X_4_	X_5_	X_6_	X_7_
I	*Ex*	5	1.5	7.5	2.5	50	90	12.5
	*En*	1.667	0.5	2.5	0.833	16.667	15	4.167
	*H*_*e*_	0.01	0.01	0.01	0.01	0.01	0.01	0.01
II	*Ex*	20	4.5	20	7.5	200	45	37.5
	*En*	3.333	0.5	1.667	0.833	33.333	5	4.167
	*H*_*e*_	0.01	0.01	0.01	0.01	0.01	0.01	0.01
III	*Ex*	45	9	28.5	55	400	20	75
	*En*	5	1	1.167	15	33.333	3.333	8.333
	*H*_*e*_	0.01	0.01	0.01	0.01	0.01	0.01	0.01
IV	*Ex*	90	18	48	150	750	5	150
	*En*	15	3	8	25	125	1.667	25
	*H*_*e*_	0.01	0.01	0.01	0.01	0.01	0.01	0.01

**Fig 3:**
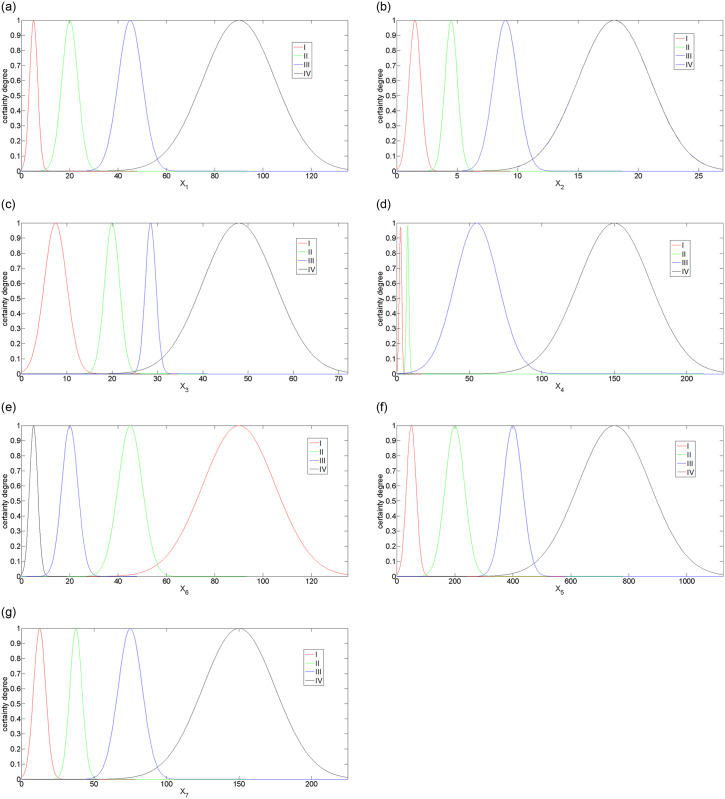
Cloud of assessment index. (a)The length ratio of the supply segmentm, (b)The longitudinal slope of the main ditch, (c)The slope of the mountain, (d)The watershed area, (e)The relative difference, (f) The vegetation coverage, (g)The daily maximum rainfall.

According to Table 3, the characters of the cloud model corresponding to different indices are calculated using the forward cloud generator, which is plotted in Fig 3.

**Fig 4:**
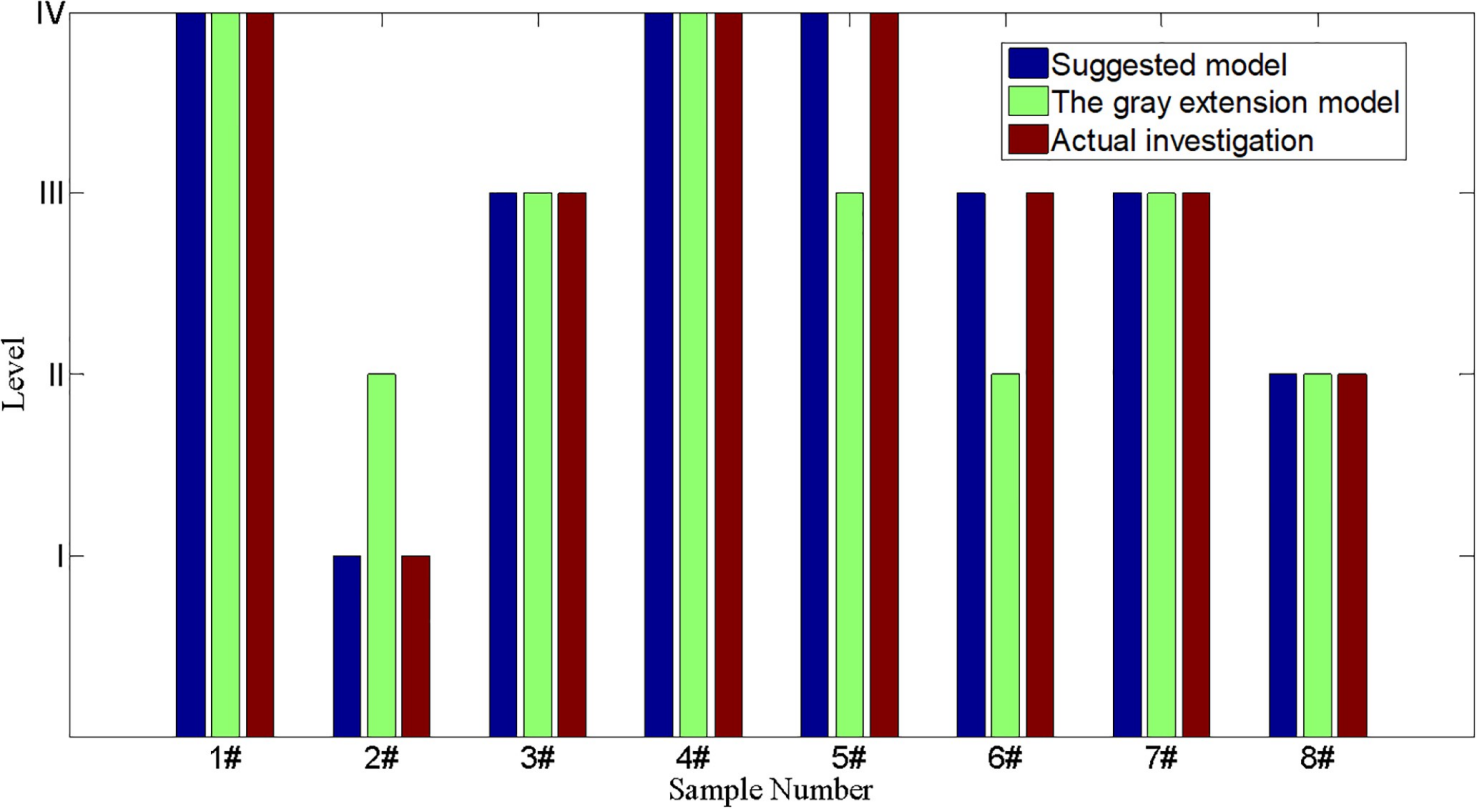
The comparison results of three methods.

Its horizontal coordinates present the magnitude of different variables; the vertical coordinates present the magnitude of certainty degree. A sub-figure in Fig 3 includes five grades: I, II, III, and IV. When a certain variable is fixed, the certainty degree of the certain point at the state grade can be obtained. The predicted result of debris flow hazard in different gullies is shown in Table 4, and compared results with the actual investigation are plotted in Fig 4.

**Table 4:** The predicted result of debris flow hazard.

Serial number	The susceptibility level of debris flow hazard				Comprehensive assessment
	I	II	III	IV	
1	0.0754	0.0998	0.0508	0.1412	IV
2	0.5362	0.0998	0.047	0.0726	I
3	0.019	0.0332	0.0711	0.0596	III
4	0	0.0707	0.0159	0.2081	IV
5	0	0	0.1607	0.1664	IV
6	0.1075	0	0.1138	0.0648	III
7	0.063	0	0.0761	0.0692	III
8	0.0881	0.1621	0	0.1507	II

The game theory combination weighting-normal cloud model is applied to evaluate the debris flow hazards. The assessment results are respectively depicted in Table 4. It can be found from Table 4 that the susceptibility levels of debris flow hazards from 1 to 8# gullies are different. The susceptibility level of debris flow hazards at 1 #, 4#, and 5# gully is IV; one at 3#, 6#, and 7# gully is III; one at 2# gully is I; one at 8# gully is II. It means that the susceptibility level of debris flow hazards at 1 #, 4#, and 5# gully is in extreme danger; one at 3#, 6#, and 7# gully is in high danger; one at 2# gully is in low danger; one at 8# gully is in medium danger, and all gullies’ qualified rate of debris flow hazards is 12.5%. So the necessary consolidation measurement should be taken to prevent debris flow hazards in other gullies except for 2# gully; for example, the steel anchor rod should be fixed in the slopes et al.

According to the comparative results of the assessment model in Fig 4, conclusions can be drawn that the results obtained by the suggested method are consistent with the actual investigation for eight different gullies. Its accuracy reaches 100% for the proposed method, which is higher than the results from the gray extension model (62.5%) [28]. So, the conclusion demonstrates that it is feasible to estimate debris flow hazards using the game theory combination weighting-normal cloud model. The method can provide more details for assessing debris flow hazards; for example, the watershed area of the 1# gully is 21.6, which should belong to level III based on Table 1. In addition, the basic membership degree of the other indicators obtained using the suggested model belongs to level IV, so the quality level probability of the 1# gully at level IV is higher than that of levels I, II, and III. So, the debris flow hazard of the 1# gully only belongs to level IV and almost impossibly belongs to levels I, II, and III. Furthermore, the susceptibility level of the 6# gully is more likely to be level III than that of the 7# gully due to the certainty degree(0.1138) of the 2# gully for group III is higher than that of the 3# gully (0.0761). The results obtained using the suggested model accurately demonstrate the susceptibility level of debris flow hazards and further determine the susceptibility grade ranking for different gullies at the same level.

## 5. Conclusions

Considering the length ratio of the supply segment (*X*_*1*_), the longitudinal slope of the main ditch(*X*_*2*_), the slope of the mountain(*X*_*3*_), watershed area(*X*_*4*_), the relative difference(*X*_*5*_), the vegetation coverage(*X*_*6*_), as well as the daily maximum rainfall(*X*_*7*_), a new evaluation method is introduced in this paper to assess the susceptibility level of debris flow hazards based on the game theory combination weighting-normal cloud model. The seven different evaluation indices are determined at first. Then, the weight coefficients of seven assessment indexes are determined based on the game theory combination weighting method. Finally, the certainty degree of other indexes are calculated using the entropy normal cloud method.

The proposed method is applied to assess the susceptibility level of debris flow; conclusions are drawn that the susceptibility level of debris flow hazards at 1 #, 4#, and 5# gully is IV; one at 3#, 6#, and 7# gully is III; one at 2# gully is I; one at 8# gully is II, so the necessary consolidation measurement should be taken to prevent debris flow hazards in other gullies except for 2# gully, for example, the steel anchor rod should be fixed in the slopes et al. Besides, the results obtained by the suggested method are entirely consistent with the actual investigation for eight different gullies; its accuracy reaches 100%, which is higher than the results from the gray extension model. The result demonstrates estimating debris flow hazards using the suggested method is feasible.

In total, the results obtained using the suggested model demonstrate the susceptibility level of debris flow hazards accurately and further determine the susceptibility grade ranking for different gullies at the same level.
